# Gastrointestinal manifestations in adult IgA vasculitis over 8.5 years: severity assessment and therapeutic outcomes

**DOI:** 10.11604/pamj.2026.54.32.52183

**Published:** 2026-06-05

**Authors:** Amira Atig, Marwa Zghidi, Yosr Bousokkaya, Nadia Ghariani, Wathek Thaljaoui, Abdelnacer Nefis

**Affiliations:** 1 Department of Internal Medicine, Farhat Hached University Hospital, Sousse, Tunisia,; 2 Intensive Care Unit, Farhat Hached University Hospital, Sousse, Tunisia,; 3 Department of Dermatology, Farhat Hached University Hospital, Sousse, Tunisia,; 4 Department of Forensic Pathology, Sidi Bouzid Hospital, Sidi Bouzid, Tunisia,; 5 Department of Digestive Surgery, Badreddine Elaloui University Hospital, Kasserine, Tunisia

**Keywords:** IgA vasculitis, Henoch-Schönlein purpura, gastrointestinal diseases, adult, corticosteroids

## Abstract

**Introduction:**

IgA vasculitis (IgAV), formerly known as Henoch-Schönlein purpura, is a small-vessel systemic vasculitis predominantly affecting children. In adults, IgAV is a rare disease; gastrointestinal (GI) involvement is nonetheless more frequent and severe than in the pediatric population, significantly impacting short-term prognosis. Data on GI manifestations in adult IgAV remain limited, and therapeutic management is not well codified.

**Methods:**

we conducted a descriptive observational study of adult patients (≥16 years) diagnosed with IgAV according to the 1990 American College of Rheumatology (ACR) or the 2012 Chapel Hill Consensus Conference (CHCC) criteria, admitted to the Internal Medicine Department of Farhat Hached University Hospital, Sousse, over the years. Gastrointestinal involvement was defined by clinical manifestations combined with at least one objective criterion (imaging or endoscopic/histological findings). Clinical and radiological severity were assessed using the Trouillier scoring system. Prolonged remission was defined as the absence of GI symptoms for at least six months after completion of corticosteroid therapy.

**Results:**

among 30 patients diagnosed with IgAV, twenty-nine patients were included in the primary analysis (mean age: 45.6±18.1 years; gender ratio: 0.87) and one patient with concurrent gastric MALT lymphoma was analyzed separately. Gastrointestinal involvement occurred in 20 patients (66.7%). The most common symptom was abdominal pain (100%), followed by vomiting (70%) and GI bleeding (40%). Severe disease was identified in 45% by clinical scoring and in 39% by radiological assessment. Corticosteroid therapy achieved prolonged remission in 63% of patients. Male gender showed a non-significant trend toward increased GI involvement (p=0.058).

**Conclusion:**

gastrointestinal manifestations in adult IgAV are frequent and often severe. The low patient accrual rate (3.5 cases/year) reflects the rarity of IgAV in adults, a disease predominantly affecting children.

## Introduction

IgA vasculitis (IgAV), previously known as Henoch-Schönlein purpura, is a small-vessel systemic vasculitis characterized by immune complex tissue deposits containing immunoglobulin A (IgA) [1,2]. This condition predominantly affects children and adolescents, with an estimated annual incidence of 10-20 cases per 100,000 children, compared to only 0.1-1.8 cases per 100,000 adults [3]. This 10- to 100-fold difference in incidence renders adult IgAV a genuinely rare disease, which has direct implications for study feasibility and sample size in single-center research.

The disease course in adults differs significantly from that in children [4]. Adult patients experience a higher incidence and greater severity of clinical manifestations, particularly gastrointestinal (GI) involvement. Short-term prognosis is largely determined by the severity of GI complications, while long-term outcomes depend on renal involvement [5,6]. Despite the clinical significance of GI manifestations in adult IgAV, management of digestive complications remains poorly codified. The existing literature on this subject is scarce, and evidence-based guidelines are not currently available [7]. This gap underscores the need for comprehensive studies examining the clinical spectrum, therapeutic approaches, and outcomes of GI involvement in adult IgAV. This study aimed to describe the clinical, therapeutic, and prognostic aspects of GI involvement in adult IgAV in a single-center cohort, with particular attention to severity assessment and treatment outcomes.

## Methods

**Study design and population:** this retrospective observational study included patients diagnosed with IgAV who were admitted to the Internal Medicine Department of Farhat Hached University Hospital, Sousse, Tunisia, between January 2014 and June 2022 (8.5 years). Patients aged 16 years or older at the time of diagnosis were included. The diagnosis of IgAV was established according to either the 1990 American College of Rheumatology (ACR) criteria or the 2012 Chapel Hill Consensus Conference (CHCC) nomenclature [1,2].

**Inclusion criteria:** age ≥16 years, confirmed IgAV diagnosis per ACR 1990 or CHCC 2012 criteria, complete medical records available, minimum follow-up of 6 months after treatment initiation.

**Exclusion criteria:** incomplete clinical records, pre-existing inflammatory bowel disease or other GI pathology that could confound assessment of GI involvement, loss to follow-up before completion of initial treatment.

**Definition of gastrointestinal involvement:** gastrointestinal involvement was defined by the presence of at least one clinical manifestation and at least one objective criterion (imaging or endoscopic/histological finding). Patients with only clinical symptoms, without objective confirmation, were not classified as having definite GI involvement.

**Clinical manifestations:** abdominal pain, vomiting, altered bowel movements, upper and/or lower GI bleeding.

**Objective confirmation (at least one required):** (1) imaging: abdominal ultrasound or CT scan evidence of GI wall thickening, intestinal ischemia, hematoma, perforation, or intussusception; (2) endoscopy/histology: leukocytoclastic vasculitis on digestive biopsy with or without IgA deposits on direct immunofluorescence.

**Severity assessment:** clinical severity was assessed using the Trouillier scoring system [7]: score ≥3 = severe; score = 2 = moderately severe; score = 0 or 1 = mild. Radiological severity was assessed by CT: severe if parietal thickening was present on multiple intestinal segments, or if ischemia, hematoma, perforation, or intussusception was identified.

### Outcome definition

**Prolonged remission:** complete absence of GI symptoms for at least six consecutive months following completion of corticosteroid tapering, without re-introduction of corticosteroids or additional immunosuppressive agents during this period.

**Special population: MALT lymphoma case:** one patient was identified as having concurrent gastric MALT lymphoma. Given the distinct clinical trajectory and combined therapeutic regimen (corticosteroids + CHOP chemotherapy), this patient was excluded from the primary efficacy analysis and is described separately as a case note.

**Statistical analysis:** data were analyzed using SPSS software (version 25.0). The Pearson chi-square test or Fisher's exact test was used for categorical variables. Student's t-test was used for continuous variables. A p-value <0.05 was considered statistically significant. Given the descriptive and exploratory nature of this study and the limited sample size inherent to a rare adult disease, all p-values should be interpreted with caution.

**Ethics approval:** this retrospective chart review was conducted in accordance with the Declaration of Helsinki. Given the retrospective nature of the study and the complete anonymization of all data before analysis, the Institutional Ethics Committee of Farhat Hached University Hospital granted an exemption from full ethical review and waived the requirement for individual informed consent. The formal exemption letter issued by the Ethics Committee (reference: 30052026) is available upon request and has been submitted to the journal as a supplementary document. All data were anonymized before analysis.

## Results

**Patient selection:** as shown in the patient flow diagram for this study (Figure 1). Of 34 patients initially identified with a diagnosis of IgA vasculitis over the 8.5-year study period, 4 were excluded: 1 due to incomplete medical records, 2 due to pre-existing gastrointestinal disease that could confound GI involvement assessment, and 1 due to loss to follow-up before treatment completion. The remaining 30 patients were included in the present study.

**Figure 1:**
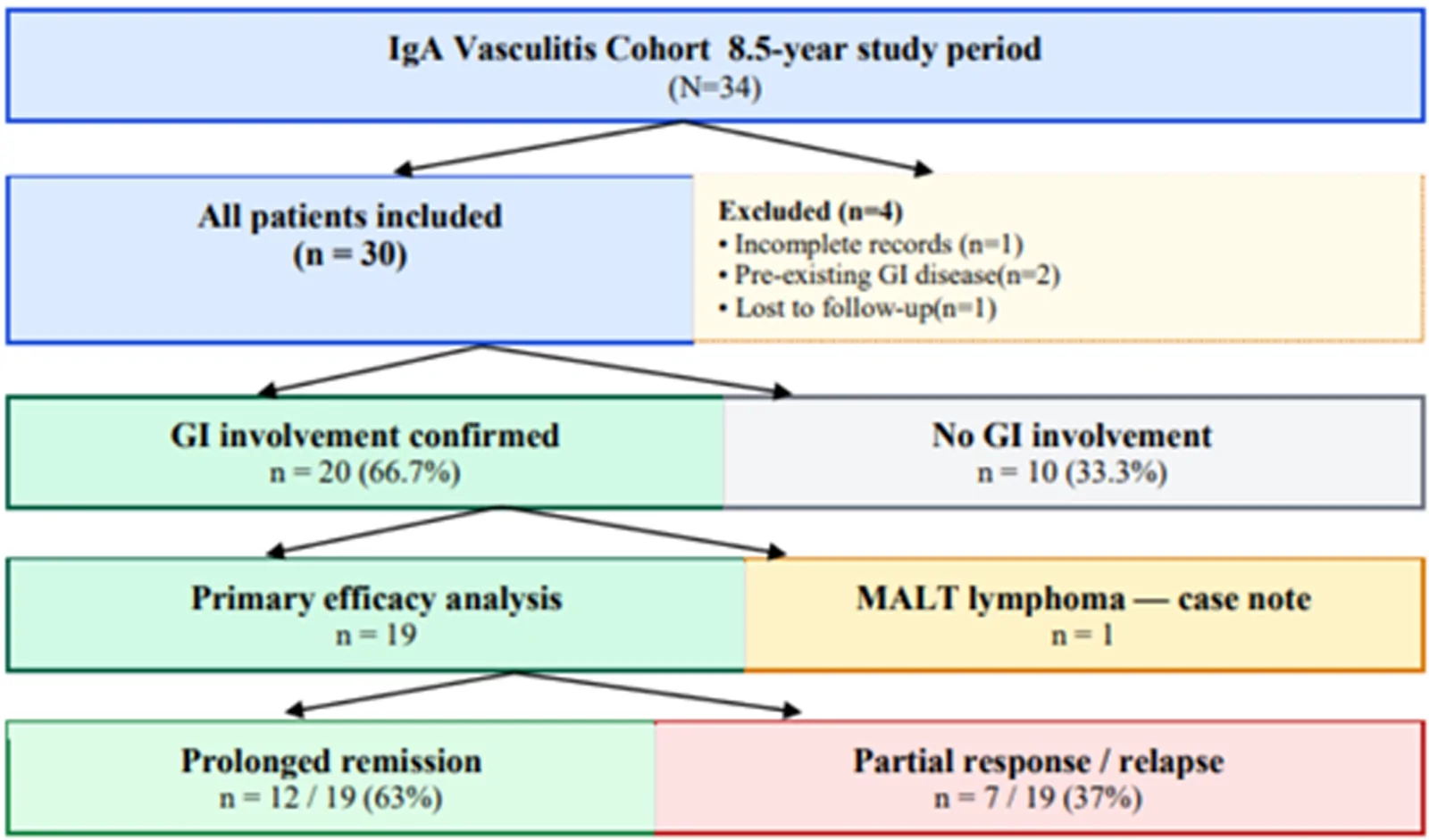
STROBE flow diagram of patient selection and outcomes; IgAV: IgA vasculitis; GI: gastrointestinal; MALT: mucosa-associated lymphoid tissue

**General population characteristics:** over the 8.5-year study period, 30 patients were diagnosed with IgAV and included in this study, corresponding to a mean accrual rate of 3.5 cases per year. The mean age at diagnosis was 45.6±18.1 years (range: 16-83 years). The cohort consisted of 14 men (46.7%) and 16 women (53.3%), with a male-to-female ratio of 0.87. A family history of autoimmune disease was identified in two patients (6.7%). Organ involvement is presented in Table 1.

**Table 1:** frequency and distribution of organ system involvement among adult IgA vasculitis patients at Farhat Hached University Hospital, Sousse, Tunisia, January 2014-June 2022 (N=30)

Characteristics	Number (n)	Percentage (%)
Gastrointestinal involvement	20	66.7
Cutaneous involvement	30	100
Articular involvement	24	80.0
Renal involvement	17	56.7

**Gastrointestinal manifestations:** gastrointestinal involvement was present in 20 patients (66.7%). All 20 patients had objective confirmation of GI involvement: 18 by CT imaging, 12 by upper GI endoscopy, and 4 by colonoscopy; 8 patients had both imaging and endoscopic confirmation. Clinical manifestations are detailed in Table 2. Abdominal pain was reported by all patients (100%), vomiting in 14 (70%), GI bleeding in 8 (40%), and altered bowel movements in 6 (30%). Hematemesis and melena each occurred in 4 cases (20%).

**Table 2:** frequency of clinical gastrointestinal manifestations among adult IgA vasculitis patients with confirmed gastrointestinal involvement at Farhat Hached University Hospital, Sousse, Tunisia, January 2014-June 2022 (N=20)

Clinical manifestation	Number (n)	Percentage (%)
Abdominal pain	20	100
Vomiting	14	70
Gastrointestinal bleeding	8	40
Hematemesis	4	20
Melena	4	20
Altered bowel movements	6	30

**Clinical and radiological severity:** using the Trouillier clinical severity score, severe digestive involvement (score ≥3) was found in 9 patients (45%), moderately severe disease (score = 2) in 6 patients (30%), and mild disease in 5 patients (25%). Severe radiological involvement was identified in 7 of 18 patients who underwent CT imaging (39%). Distribution is presented in Table 3.

**Table 3:** clinical and radiological severity of gastrointestinal involvement assessed by the Trouillier scoring system and CT imaging among adult IgA vasculitis at Farhat Hached University Hospital, Sousse, Tunisia, January 2014-June 2022 (N=20)

Severity category	Number (n)	Percentage (%)
Clinical: severe (score ±3)	9	45
Clinical: moderately severe (score = 2)	6	30
Clinical: mild (score = 0 or 1)	5	25
Radiological: severe (CT, n=18)	7	39

**Endoscopic and histological findings:** upper GI endoscopy was performed in 12 of 20 patients (60%). The most common findings were mucosal erythema (75% of those scoped), petechial purpura (60%), and mucosal erosions (50%). Colonoscopy was performed in 8 patients, revealing abnormal findings in 4 (50%), predominantly circumscribed erythematous lesions of 3-10 mm. Direct immunofluorescence demonstrated IgA deposits in 2 patients (10% of all GI patients; 17% of those biopsied). These findings are summarized in Table 4.

**Table 4:** endoscopic and histological findings in adult IgA vasculitis patients with gastrointestinal involvement undergoing upper gastrointestinal endoscopy, colonoscopy, and mucosal biopsy at Farhat Hached University Hospital, Sousse, Tunisia, January 2014-June 2022

Finding	Patients examined (n)	Abnormal findings n (%)
Upper GI endoscopy	12	12 (100)
Mucosal erythema		9 (75)
Petechial purpura		7 (60)
Mucosal erosions		6 (50)
Colonoscopy	8	4 (50)
Histology (DIF: IgA deposits)	12	2 (17)

DIF: direct immunofluorescence; GI: gastrointestinal

**Associated conditions:** *Helicobacter pylori* infection was identified in 4 patients (20%). All received eradication therapy. Complete resolution of cutaneous and gastric manifestations occurred in 1 patient following eradication alone. The remaining 3 patients required additional corticosteroid therapy; 2 achieved prolonged remission, and 1 had a partial response.

**Case note: gastric MALT lymphoma:** one 43-year-old female patient with IgAV and GI involvement was found to have concurrent gastric MALT lymphoma on endoscopic biopsy. *H. pylori* infection was confirmed. She received combined prednisone (0.5 mg/kg for 6 months) and CHOP chemotherapy, achieving complete histological and clinical remission at 12-month follow-up. This patient was excluded from the primary treatment outcome analysis.

**Treatment and outcomes:** corticosteroid therapy was administered to all 19 patients in the primary efficacy analysis. The typical regimen was prednisone 0.5-1 mg/kg/day with gradual tapering over 8-12 weeks. Prolonged remission (absence of GI symptoms for ≥6 months after treatment completion) was achieved in 12 patients (63%). Seven patients (37%) experienced persistent symptoms or relapse requiring treatment adjustment.

**Predictive factors:** gastrointestinal involvement was numerically more frequent in male patients (p=0.058), but this difference did not reach statistical significance. No significant association was identified between GI involvement and cutaneous purpura distribution, renal involvement, or age at diagnosis.

## Discussion

This single-center descriptive study provides data on GI involvement in adult IgAV over the years. The low accrual rate of 3.5 cases/year, yielding only 30 patients over 8.5 years, itself constitutes an important finding, directly reflecting the rarity of IgAV in the adult population. The GI involvement occurred in 20 patients (66.7%). The most common symptom was abdominal pain (100%), followed by vomiting (70%) and GI bleeding (40%). In addition, the current study finds that male gender showed a non-significant trend toward increased GI involvement (p=0.058).

**Clinical manifestations and severity:** the predominance of abdominal pain (100%), high frequency of vomiting (70%), and GI bleeding (40%) confirms the significant morbidity of GI involvement in adult IgAV. Systematic application of the Trouillier score allowed objective disease stratification. Our severity distribution is broadly consistent with the original Trouillier cohort [7], which reported severe disease in 66.7%. The more severe GI phenotype in adults compared to children is well recognized and may relate to higher comorbidity burden, delayed diagnosis, or age-related differences in immune regulation [4,8].

**Imaging and endoscopic evaluation:** computed tomography imaging identified severe radiological involvement in 39% of patients who underwent imaging. The characteristic features of intestinal wall thickening and mesenteric edema facilitated early diagnosis [5,9]. Upper GI endoscopy and colonoscopy findings in our cohort, including mucosal erythema, petechial purpura, and erosions, are consistent with those reported in previous series [10,11]. The limited histological yield from endoscopic biopsies, IgA deposits confirmed in only 17% of biopsied patients, reflects the superficial nature of most endoscopic biopsies and the patchy distribution of vasculitic lesions [12,13].

**Therapeutic considerations:** prolonged remission was achieved in 63% of patients, consistent with the efficacy of corticosteroids in GI IgAV reported in both pediatric and adult studies [14,15]. However, the absence of a control group and retrospective design limit causal inference. The 37% rate of partial response or relapse highlights the need for steroid-sparing alternatives in refractory disease.

**Helicobacter pylori and associated malignancy:** *H. pylori* was identified in 20% of patients. The association between *H. pylori* infection and IgAV has been previously described, with several reports suggesting that eradication may contribute to disease resolution in a subset of patients [16,17]. In our cohort, eradication alone was sufficient in only one case, suggesting a limited independent role in disease maintenance in most patients. The MALT lymphoma case illustrates the importance of systematic malignancy screening in adult IgAV, particularly in patients over 40 years of age [18-20].

**Predictive factors:** the non-significant trend toward increased GI involvement in male patients (p=0.058) is consistent with observations by Hočevar *et al*. [21] but remains hypothesis-generating in our cohort. Several biomarkers, including D-dimer [22], neutrophil-to-lymphocyte ratio [23], and fecal calprotectin [24] have been proposed as predictive markers and warrant prospective evaluation.

**Study limitations:** this study has several limitations that must be acknowledged. Most fundamentally, the sample size of 30 patients, despite an 8.5-year collection period, is small for a single-center study. This low accrual rate is not a methodological limitation but rather an inherent epidemiological constraint: IgAV is predominantly a disease of childhood, with an estimated annual incidence of 10-20 per 100,000 in children and only 0.1-1.8 per 100,000 in adults [3]. This condition makes it nearly impossible to achieve large sample sizes in a single-institution study without multi-year follow-up. Our accrual rate of 3.5 cases/year is consistent with the published epidemiology of adult IgAV and reflects the true rarity of this condition rather than a recruitment failure.

As a direct consequence of this small sample size, the multivariable analysis is underpowered. The absence of statistically significant predictors, including the male gender trend (p=0.058) should be interpreted as reflecting insufficient power rather than a true absence of association. A formal post-hoc power calculation confirms that a sample of 20 patients with GI involvement provides less than 40% power to detect a moderate effect size at alpha=0.05. Additional limitations include the retrospective single-center design, which introduces selection bias and limits generalizability. These limitations underscore the critical need for multicenter and international collaborative registries to study adult IgAV. Pooling data across multiple centers, ideally within a prospective registry framework, is the only feasible approach to achieving the sample sizes required for robust prognostic and therapeutic studies in this rare adult disease.

## Conclusion

In this single-center descriptive series spanning 8.5 years, the accrual of only 30 adult patients with IgAV confirms that IgAV is a rare disease in adults despite its relative frequency in children. GI manifestations occurred in approximately two-thirds of patients and were frequently severe by standardized scoring. Systematic use of clinical and radiological severity scores facilitated therapeutic decision-making. Corticosteroid therapy achieved prolonged remission in 63% of patients. The male gender showed a non-significant trend toward increased GI involvement requiring validation in larger cohorts.

### What is known about this topic

- IgA vasculitis (Henoch-Schönlein purpura) is the most common systemic vasculitis in children, but its presentation in adults is rare and more severe;
- Gastrointestinal involvement occurs in approximately 50-75% of IgA vasculitis cases and can manifest as abdominal pain, gastrointestinal bleeding, and in rare cases, life-threatening complications such as bowel perforation;
- Current management of gastrointestinal manifestations in IgA vasculitis relies on corticosteroids, though evidence regarding optimal therapeutic strategies in adults remains limited.

### What this study adds

- This study provides a descriptive analyses of gastrointestinal manifestations in adult IgA vasculitis over an 8.5-year period, contributing to the limited literature on this age group;
- It characterizes the spectrum of gastrointestinal severity in adults and identifies clinical and biological factors associated with severe gastrointestinal involvement;
- It describes real-world therapeutic outcomes, offering practical insights into the management of gastrointestinal complications in adult IgA vasculitis in a North African clinical setting.
